# Phylogenetic Analyses of *Shigella* and Enteroinvasive *Escherichia coli* for the Identification of Molecular Epidemiological Markers: Whole-Genome Comparative Analysis Does Not Support Distinct Genera Designation

**DOI:** 10.3389/fmicb.2015.01573

**Published:** 2016-01-19

**Authors:** Emily A. Pettengill, James B. Pettengill, Rachel Binet

**Affiliations:** ^1^Division of Microbiology, Office of Regulatory Science, U.S. Food and Drug Administration, Center for Food Safety and Applied Nutrition College Park, MD, USA; ^2^Division of Public Health Informatics and Analytics, Office of Analytics and Outreach, U.S. Food and Drug Administration, Center for Food Safety and Applied Nutrition College Park, MD, USA

**Keywords:** *Shigella*, enteroinvasive *E. coli* (EIEC), phylogeny, whole genome sequencing, classification, epidemiological markers

## Abstract

As a leading cause of bacterial dysentery, *Shigella* represents a significant threat to public health and food safety. Related, but often overlooked, enteroinvasive *Escherichia coli* (EIEC) can also cause dysentery. Current typing methods have limited ability to identify and differentiate between these pathogens despite the need for rapid and accurate identification of pathogens for clinical treatment and outbreak response. We present a comprehensive phylogeny of *Shigella* and EIEC using whole genome sequencing of 169 samples, constituting unparalleled strain diversity, and observe a lack of monophyly between *Shigella* and EIEC and among *Shigella* taxonomic groups. The evolutionary relationships in the phylogeny are supported by analyses of population structure and hierarchical clustering patterns of translated gene homolog abundance. Lastly, we identified a panel of 254 single nucleotide polymorphism (SNP) markers specific to each phylogenetic cluster for more accurate identification of *Shigella* and EIEC. Our findings show that *Shigella* and EIEC are not distinct evolutionary groups within the *E. coli* genus and, thus, EIEC as a group is not the ancestor to *Shigella*. The multiple analyses presented provide evidence for reconsidering the taxonomic placement of *Shigella*. The SNP markers offer more discriminatory power to molecular epidemiological typing methods involving these bacterial pathogens.

## Introduction

*Shigella* species are a leading cause of bacterial diarrhea (Walker et al., 2010). Worldwide, it is estimated that 164.7 million people are infected by *Shigella* annually (495,000 of those people in the United States) often through contaminated food and water (Scallan et al., 2011). Enteroinvasive *Escherichia coli* (EIEC), like *Shigella*, can also cause dysentery-like symptoms (Taylor et al., 1988). *Shigella* and EIEC are, in essence, strict human pathogens, sharing similar pathogenic mechanisms but their evolutionary relationship on a genomic level has not been determined. Although, the close relationship between *Shigella* and *E. c*oli has been acknowledged since 1898 (reviewed by Lan and Reeves, 2002), in the 1940s Ewing proposed classifying the four species in the new genus *Shigella* (*S. dysenteriae*, *S. flexneri*, *S. boydii*, and *S. sonnei*) based on the antigen characteristics of those species (Edwards and Ewing, 1986). Since that time, numerous studies have indicated that the phylogenetic history does not support this current classification (Pupo et al., 2000; Lan and Reeves, 2002; Escobar-Páramo et al., 2003; Lan et al., 2004; Sahl et al., 2015).

Volunteer feeding studies have shown that whereas 10 to a few 100 *Shigella* cells were enough to cause illness in healthy adults, the infective dose for three different EIEC strains was more in the 10^8^ range, justifying the need for clinical medicine to maintain two separate genera (DuPont et al., 1971; Mathewson et al., 1985). However, considering that most governmental health agencies do not currently require reporting EIEC infections, their impact on diarrheal disease and their genetic diversity is not well-understood. The recent involvement of EIEC O96:H19 as the source of outbreaks severely affecting healthy individuals in Italy, Great Britain and a case reported in Spain illustrates that EIEC can be a potential threat to public health and provides new motivation for improving our understanding of EIEC for rapid and accurate identification (Escher et al., 2014; Michelacci et al., 2015; Pettengill et al., 2015). This new motivation is reinforced by a long established need to understand the evolutionary relationships between *Shigella*, EIEC and non-invasive *E. coli* for improved detection and surveillance.

Traditional microbiology differentiates *Shigella* from *E. coli* based on their physiological and biochemical characteristics, with EIEC being more metabolically active than *Shigella* (Edwards and Ewing, 1986). Sero-agglutination assays are afterward generally performed for the differentiation of members of the genus *Shigella*, but cross-reactivity with certain EIEC serotypes have been observed (Liu et al., 2008). Developing nucleic acid-based detection methods combining higher discriminatory power with low limits of detection are ideal but rely on the availability of suitable markers based on a wide diversity of isolates for that organism (Zhao et al., 2014). Currently, most molecular assays for the diagnosis of *Shigella* rely mainly on targeting the large ∼220-kbp invasive plasmid that is also shared by EIEC and, hence, cannot differentiate between the pathogens (Binet et al., 2014). Although, two recent studies proposed PCR assays to distinguish between *Shigella* species (Sahl et al., 2015) or between *Shigella* and EIEC (Pavlovic et al., 2011), the first study did not include any EIEC in their exclusivity panel and the second study included only 18 isolates of *Shigella* and 11 isolates of EIEC in their inclusivity panel.

In this study, we studied the evolutionary relationships among a wide diversity of strains that represent the *Shigella* genus and closely related EIEC. Comprehensive phylogenetic analyses were performed to determine if *Shigella* and EIEC are distinct evolutionary groups. Genome similarity was then investigated using a Bayesian clustering method that does not impose the bifurcating structure of phylogenetic analyses. Samples were then hierarchically clustered based on differences in abundance of predicted protein homologs to determine functional genomic differences. Lastly, we identified single nucleotide polymorphisms (SNPs) that were diagnostic of different phylogenetic clades that could be used to type and/or discriminate among those lineages.

## Materials and Methods

### Growth of Strains, DNA Isolation, and Genome Retrieval

Pure culture isolates for 33 *Shigella* and *E. coli* strains (**Supplementary Table S1**) were grown from frozen stocks on Trypticase Soy Agar plates and incubated overnight at 37°C. A minimum of three colonies were then inoculated into either *Shigella* Broth (if *Shigella* sp.; Center for Food Safety and Applied Nutrition, 2001) or Trypticase Soy Broth (if EIEC strains) for DNA extraction after overnight growth at 37°C. Genomic DNA was extracted using DNeasy^®^ Blood and Tissue kits (QIAGEN, Valencia, CA, USA) according to manufacturers’ instructions. An additional 80 genomes (**Supplementary Table S1**) were retrieved in June 2014 from the NCBI SRA database using the SRA Toolkit v. 2.3.5-2 in fastq format^1^. Assembled genomes from Sahl et al. (2015) were retrieved from NCBI in February 2015.

### Library Construction, Genome Sequencing, and Sequence Data

DNA was quantified using the Qubit^®^ 2.0 Fluorometer and the Qubit^®^ HS Assay kit (Life Technologies, Foster City, CA, USA). Samples were diluted to 0.2 ng/μl and stored at -20°C until library preparation. Libraries were prepared using the Nextera XT DNA Sample Preparation Kit (Illumina^®^, San Diego, CA, USA). Sequencing reactions were performed with the MiSeq v2 chemistries with 250 bp paired-end read lengths and a 500-cycle cartridge and processed on a MiSeq platform (Illumina^®^, San Diego, CA, USA) to obtain data in fastq format. All the sequencing data generated for this project are available through bioproject accessions PRJNA273284 and PRJNA230969 at the National Center for Biotechnology Information (NCBI).

### Quality Control, Trimming, and Genome Assembly

Reads were trimmed and low quality bases (Q-scores < 20) filtered using the DynamicTrim program in SolexaQA v. 2.2 (Cox et al., 2010). Trimmed reads were then assembled using SPAdes v. 3.1.1 (Bankevich et al., 2012) with default settings. To ensure that assemblies were of high quality (e.g., low number of contigs and adequate total length), we obtained assembly statistics using the program Quast (Gurevich et al., 2013; **Supplementary Table S3**). Using the *de novo* assemblies from SPAdes, SNP matrices were produced using the reference-free approach implemented in kSNP v2.0 (Gardner and Hall, 2013). For the kSNP analyses we used a *k*-mer value of 21, which was identified as the best fitting value based on the auxiliary script kChooser provided with that software.

Although, kSNP produces three matrices (composed of “all,” “majority,” and “core” SNPs), we focused on the core matrix as it is a more conservative method for identifying variant sites and better suited to remove recombination/horizontally transferred genomic elements form the analysis. The core matrix contains no missing data meaning there is a nucleotide state at each position in the alignment for all individuals. For kSNP analyses that included the *Salmonella* genomes, a total of 660,234 SNPs were identified and the number of core SNPs was 2,348. Analyses without *Salmonella* genomes had a total of 598,876 SNPs and 7,062 core SNPs. Of the core SNPs, 385 (16%) including *Salmonella* genomes and 1556 (22%) excluding *Salmonella* genomes were homoplastic (non-informative) SNPs. The proportions of homoplastic are lower than other kSNP analyses of *E. coli* genomes (37.6%; Gardner and Hall, 2013).

### Serotyping

*Shigella* species are routinely serotyped with Statens Serum Institute species specific pool antisera (Cedarlane, Burlington, NC, USA) upon reception and by an in-house multiplex PCR assay (Binet, personal communication). Although the serotype is also confirmed with serotype specific Denka Seiken agglutinating sera (Thermo Fisher Scientific, Lenexa, KS, USA) on a case-by case basis, we did not confirm the identity of the nine *Shigella* isolates we sequenced in this study at the serotype level since they came from reputable bacterial collections, i.e., ATCC and CDC (**Supplementary Table S1**). All EIEC strains we sequenced were, however, conventionally serotyped with polyclonal O antigens from Statens Serum Institute (Cedarlane, Burlington, NC, USA) using a boiling method detailed by the manufacturer. For the additional genomes added to the study, in the absence of isolates, pertinent information was obtained directly from NCBI as provided at the time of submission or from associated publications when available (Holt et al., 2012; Escher et al., 2014; Sahl et al., 2015).

### Phylogenetic Analysis and Sample Labeling Designations

Using the core matrix produced by kSNP, phylogenetic inference analysis was performed using GARLI (Genetic Algorithm for Rapid Likelihood Inference) v. 2.0.1019 under the GTR + I + Γ model and other default settings; trees were visualized with Figtree v. 1.3.1 (Zwickl, 2006; Rambaut and Drummond, 2009). To estimate the best topology based on the observed data, we ran 1000 replicate analyses and present the tree with the highest likelihood value. To estimate topological support for the different relationships, we ran 1000 bootstrap replicates that were then summarized using the SumTrees utility within the DendroPy package (Sukumaran and Holder, 2010). We chose not to remove homoplasious sites because bacterial phylogenetic topologies have been shown to be robust to the inclusion of such sites and removing them may in fact be detrimental to estimates of branch length (Hedge and Wilson, 2014).

*Escherichia coli* strains present in the phylogenetic tree are listed by the type of *E. coli*, the O antigen and H antigen (if known) followed by strain replicate number in parentheses. Other abbreviations found in the tree are: EIEC: enteroinvasive *E. coli*; EAEC: enteroaggregative *E. coli*; STEC: Shiga-toxin producing *E. coli*; ExPEC: extraintestinal pathogenic *E. coli*; EPEC: enteropathogenic *E. coli*; EHEC: enterohemorrhagic *E. coli* (Clements et al., 2012). *Shigella* strains are designated by genus and species, serotype (if known) followed by strain replicate number in parentheses. Abbreviations for *Shigella* species are as follows: SD: *S. dysenteriae*; SF: *S. flexneri*; SB: *S. boydii*, SS*: S. sonnei*.

### Diagnostic SNP Detection

A separate kSNP analysis was performed without the two *Salmonella* outgroup samples to obtain a core SNP matrix for only *Shigella* and EIEC samples (described above). A custom python script was used with the core matrix to identify those SNPs that were specific to the groups from the SNP-based phylogeny. We define a diagnostic SNP as a position in the core matrix where the nucleotide state is the same among all members of a group and that state differs from all non-members. For each diagnostic SNP (**Supplementary Table S2**), we report the SNP nucleotide region of 21 bp (or *k*-mer), the diagnostic SNP state of that cluster, the position in relation to a reference genome (SD serotype 1, NCBI: CP000034), the name of the gene (if applicable), the product (if applicable), the functional Clusters of Orthologous Groups of proteins (COG) category and the reference genome locus tag (if applicable).

### STRUCTURE Analyses

The STRUCTURE program performed model-based Bayesian clustering of genomes using the core SNP matrix without *Salmonella*, *E. fergusonii* or SB serotype 13 (related to *E. albertii*; Pritchard et al., 2000; Falush et al., 2003). Default parameters that consider admixture, were run for values of *k* between 2 through 11. The best fitting value of *k* identified by STRUCTURE HARVESTER based on changes in likelihood scores across the values of *k* as well as results from the value of *k* corresponding to the number of phylogenetic cluster (Evanno et al., 2005; Earl and vonHoldt, 2012). We ran ten replicate STRUCTURE runs for *k* = 2 to 11, each consisting of 6 × 10^4^ generations, the first 10^4^ served as the burn-in. Analyses were visualized using the DISTRUCT program (Rosenberg, 2004).

### Genome Annotation, Homology Prediction, and Similarity Matrix

**FIGURE 1:**
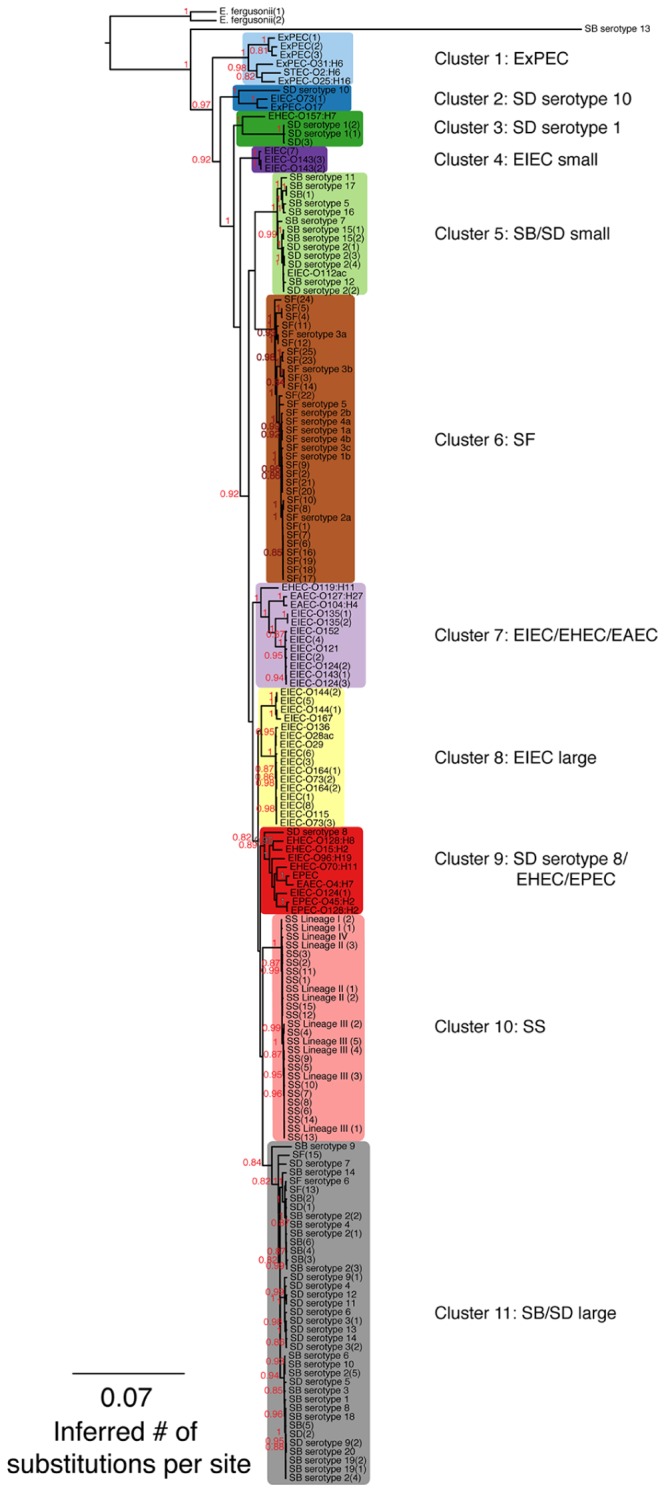
A maximum-likelihood (ML) phylogeny of *Shigella*, enteroinvasive *Escherichia coli* (EIEC) and non-invasive *E. coli* strains based on 7,062 core SNPs using kSNP (Gardner and Hall, 2013). The ML tree was generated using GARLI v. 2.0.1019 under the GTR + I + Γ model and other default settings (Zwickl, 2006). Trees were visualized with Figtree v. 1.3.1 (Rambaut and Drummond, 2009). The best tree was chosen from 1,000 runs of the data set and bootstrap values (1,000 iterations) are reported above each node. Bootstrap values <80% are not shown. A tree that includes the *Salmonella* outgroup can be found in **Supplementary Figure S1**.

Genome annotation was performed with RAST v. 2.0 (ClassicRAST; Overbeek et al., 2013). Annotated genomes were used to predict the homology of predicted proteins using the GET_HOMOLOGUES (Contreras-Moreira and Vinuesa, 2013) program which uses a BLASTP bidirectional best hit approach with the following parameters: 75% amino acid sequence coverage, 1e-05 E-value and 60% sequence identity. This produced an abundance matrix of 3,777 predicted protein homologs that were identified in at least two genomes. Manhattan distances were calculated from this matrix and clustered using the average linkage method with the hclust function in R Core Team (2014). Hierarchical clusters are colored to match the phylogenetic clusters in **Figure 1** in a bar next to the heat map. To obtain bootstrap probabilities (BPs) for the dendrogram and assign approximately unbiased *p*-values (AU), the Pvclust program in R was used with 10,000 replicates and shown next to a heat map generated with ggplot2 (Suzuki and Shimodaira, 2006; Wickham, 2009; R Core Team, 2014).

### Antibiotic Resistance-Related Annotation and Hierarchical Clustering

Using all genomes except those from Sahl et al. (2015) study, antibiotic resistance, the genes of antibiotic targets and biosynthesis genes were determined from a local BLASTN search using files available from the Comprehensive Antibiotic Resistance Database (downloaded in January 2015) with parameters set to an E-value of 1e-06 and 75% identity (**Supplementary Figure S4**; McArthur et al., 2013). The data were filtered to include genes that were present in at least two genomes. Hierarchical clustering, bootstrap support and approximately unbiased *p*-values were determined as described above.

### Evaluation of Previously Described Molecular Assays for the Differentiation of *Shigella* and EIEC

Sahl et al. (2015) reported 11 primer pairs that were specific to their phylogenetic analysis of *Shigella* but they did not include EIEC strains in their analysis. Similarly, Pavlovic et al. (2011) reported that primers targeting the β-glucuronidase gene (*uidA*) and the lactose permease gene (*lacY*) could differentiate 18 isolates of *Shigella* from 11 isolates of EIEC. The primers sequence identities from those two studies were examined, *in silico*, using local BLAST searches against the 169 genomes in our analyses. In **Supplementary Figures S5** and **S6**, genomes for which the particular primer pair exhibited 95% or greater, and 92% or greater similarity, respectively, were shown in blue to predict PCR amplification. The figures were made using R Core Team (2014).

## Results

### Phylogeny

One hundred and seventy-one genomes were selected to encompass a large selection of EIEC strains and represent the diversity of the *Shigella* genus. Genomes from 35 isolates were inhouse sequenced draft genomes while 136 were available in public databases (**Supplementary Table S1**). We used 23 isolates of SD, including a minimum of 14 serotypes, 36 SF isolates, including at least six serotypes, 32 SB isolates, covering all 20 serotypes, 26 SS isolates, 32 EIEC isolates with 15 different serotypes, 18 isolates of non-invasive *E. coli* composed of 14 different serotypes, two isolates of *E. fergusonii.* The genomes of two *Salmonella* isolates were used for an outgroup (**Table 1**).

**Table 1:** Number of bacterial isolates and serotypes.

Tree label	Description	Isolates	Serotypes
EIEC	Enteroinvasive *E. coli*	33	15
EAEC	Enteroaggregative *E. coli*	3	3
STEC	Shiga-toxin producing *E. coli*	1	1
ExPEC	Extraintestinal *E. coli*	6	3
EPEC	Enteropathogenic *E. coli*	3	2
EHEC	Enterohemorrhagic *E. coli*	5	5
*E. fergusonii*	*E. fergusonii*	2	1
SD	*Shigella dysenteriae*	23	14
SF	*Shigella flexneri*	36	6
SB	*Shigella boydii*	32	20
SS	*Shigella sonnei*	26	1
*S. enterica*	*Salmonella enterica*	2	1

	Total	171	72

Single nucleotide polymorphisms found in every genome, defined as core SNPs, were used to generate SNP matrices. The kSNP v. 2.0 program (Gardner and Hall, 2013), which uses a *k*-mer based approach to identify variant sites across a set of genomes, generated SNP matrices consisting of 7,062 or 2,348 core SNPs depending on whether the *Salmonella* outgroup was excluded (**Figure 1**) or included (**Supplementary Figure S1**). Subsequent phylogenetic reconstruction based on both SNP matrices resolved 11 groups that did not follow the taxonomic classification of the samples, thus implying that *Shigella*, EIEC, and non-invasive *E. coli* were polyphyletic (**Figure 1**; **Supplementary Figure S1**). With the exception of the EIEC large cluster, all clusters had adequate bootstrap support (greater than 0.83). The phylogeny shows that SD serotype 1, SD serotype 8, SD serotype 10, and SB serotype 13 do not cluster with any other *Shigella* serotypes (**Figure 1**). Clusters 1, 2, 3, 7, and 9 were composed of either EIEC or *Shigella* strains in combination with non-invasive *E. coli* strains, whereas clusters 4, 5, 6, 8, 10, and 11 contained only EIEC or *Shigella*. Clustering of SB and SD genomes suggests there are not distinct SB and SD lineages. Most SF genomes clustered together except those of SF serotype 6 that falls into cluster 11 with several serotypes of SB and SD. In the absence of actual isolates for SF(13) and SF(15) to conventionally determine their O-antigen type by sero-agglutination, we turned to molecular serotyping targeting the *wzx* and *wzy* genes involved in the assembly of the O-antigen. Gene alignments between SF(13) and SF(15) and *S*. *flexneri* serotype 6 *wzx* and *wzy* genes were 99% homolog (data not shown) and both strains identified as *E. coli* O147, which is nearly identical to *S. flexneri* type 6 (Liu et al., 2008), using the SerotypeFinder software (v. 1.1) accessible on the Center for Genomic Epidemiology server^2^. For perspective on how many SNP differences are represented by the branch lengths, histograms of the pairwise distances of total SNP number between pairs of genomes can be found in **Supplementary Figure S2**.

### Population Structure of SNP Clustering

**FIGURE 2:**
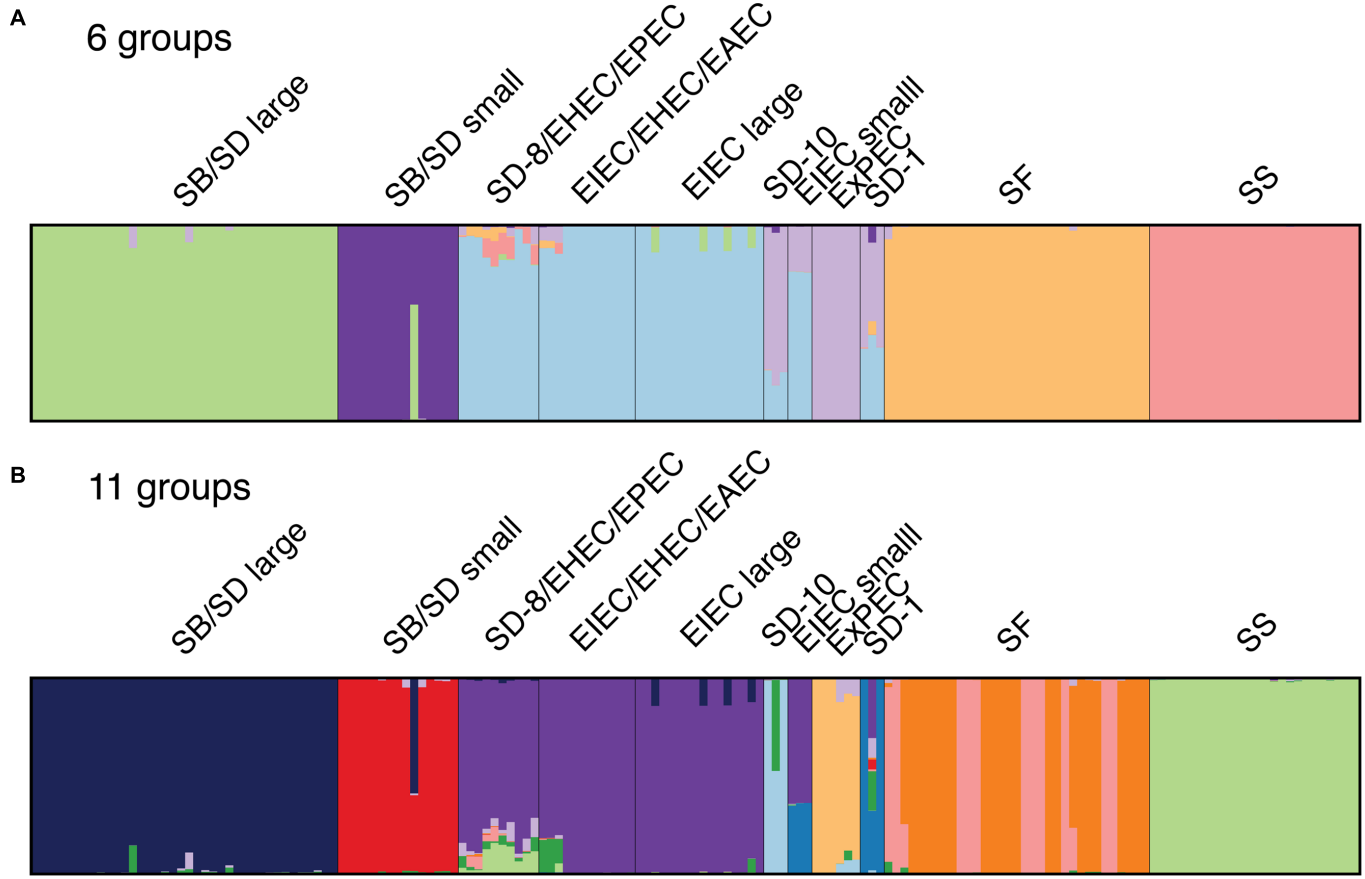
DISTRUCT diagrams showing clustering of *Shigella*, EIEC and non-enteroinvasive *E. coli* genomes derived from the STRUCTURE analyses with the core SNP matrix: (A) six genetic groups were determined to be the best fitting number of groups by STRUCTURE HARVESTER program and (B) the 11 groups identified from the phylogeny. Each bar represents a single genome and the color represents the proportion of SNPs that represent a cluster. Color assignment was random and does not coordinate with phylogenetic clusters in **Figure 1**.

Genome similarity was then investigated using a Bayesian clustering method that does not impose the bifurcating structure of phylogenetic analyses. The population structure of the samples was therefore examined using the Bayesian model-based program STRUCTURE v. 2.3.4. the core SNP matrix from the kSNP program without *Salmonella* as input. The program assigns individuals to a fixed number of clusters (*k*) allowing for admixture (e.g., recombination, ancestral polymorphism, horizontal gene transfer). The program STRUCTURE Harvester was used to infer the optimal value of *k* that best fits the data (Evanno et al., 2005; Earl and vonHoldt, 2012), which was determined to be 6 (**Figure 2A**). We also chose a *k* value of 11 to represent the number of clusters in the phylogenetic analyses (**Figures 1** and **2B**). Both cluster schemes were similar to the phylogeny, particularly for SS, SF, ExPEC, and EIEC lineages and the two distinct SB/SD lineages (**Figure 1**). Genomes in clusters that include SD serotype 1, SD serotype 10, and SD serotype 8 shared core SNPs with genomes in the EIEC, ExPEC and very small proportions of SF and SS clusters (**Figures 2A,B**). When core SNPs from SF genomes were grouped into 11 genetic groups, the phylogeny topology was similar to that of the six groups with the exception of the SF genomes which appear to have two genetic backgrounds and these roughly correspond to the clustering observed in the phylogeny (**Figure 1**; **Supplementary Figure S3**).

### Clusters of Predicted Protein Homologs

**FIGURE 3:**
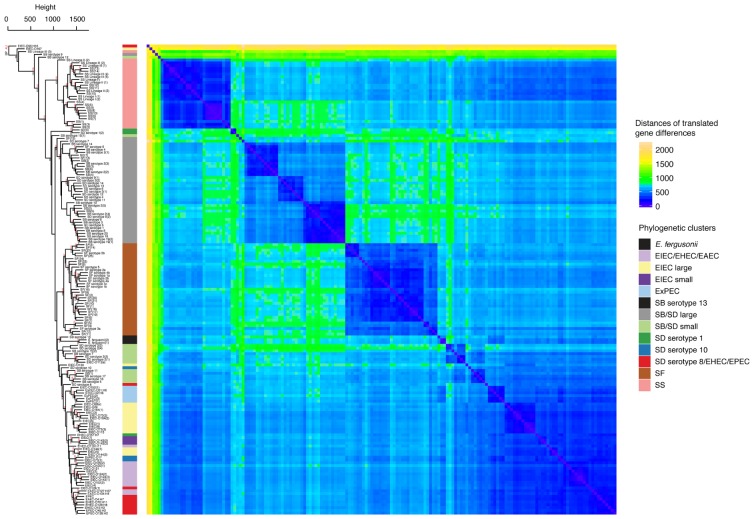
Hierarchical clustering and heat map illustrating the differences in predicted protein homologs between genomes. Manhattan distances were calculated from a pairwise abundance matrix of 3,777 predicted protein homologs that were identified using the default BLASTP bidirectional best hit approach (75% amino acid sequence coverage, 1e-05 E-value and 60% sequence identity) within the program GET_HOMOLOGUES (Contreras-Moreira and Vinuesa, 2013). Only genes shared by at least two samples were included. Blue cells on the heat map indicate that genomes share more similar genes. The dendrogram on y-axis indicates hierarchical clustering of the abundance matrix using the average linkage method and Manhattan distances with bootstrap probabilities (BP, only values of ≥80 shown in black) and approximately unbiased *p*-values (AU, only values of ≥95 shown in red) from 10,000 replicates. The phylogenetic group of each genome from **Figure 1** is represented as a colored bar in between the dendrogram and the heat map.

The differences between the gene content of the genomes was then investigated based on the abundance of predicted protein homologs. After annotating all genomes with RAST (Overbeek et al., 2013), homologous translated genes were identified using the program GET_HOMOLOGUES which uses a BLASTP bidirectional best-hit approach (Contreras-Moreira and Vinuesa, 2013). While restricting our analyses to the genes that were shared between at least two individuals, we obtained a matrix composed of 3,777 genes and their abundances within each genome. The abundance matrix was hierarchically clustered with the average linkage method and Manhattan distances to identify differences in these profiles using the R package Pvclust (Suzuki and Shimodaira, 2006; R Core Team, 2014). Pvclust was also used to obtain statistical support for clusters based on both AU *p*-values and BP (Suzuki and Shimodaira, 2006). This showed that genomes from the phylogeny in the SS, SF, and SB/SD large clusters have significantly clustered translated gene abundance profiles with BP and AU of 100/100, 100/100, and 93/97, respectively (**Figure 3**). Hierarchical clustering of antibiotic resistance related genes shows patterns that are consistent with these studies and may indicate lineage specific selection in SS and some SD serotypes (**Supplementary Figure S4**).

### Lineage-Specific SNP Identification and Evaluation of Previously Described Molecular Assays for the Differentiation of *Shigella* and EIEC

To identify lineage specific SNPs, we excluded the *Salmonella* outgroup to focus on differentiating among *Shigella* and EIEC lineages. From 7,062 core SNPs, we found 254 SNP positions that were diagnostic for each of the clusters (**Supplementary Table S2**). A description of the diagnostic SNPs by phylogenetic cluster is found in **Table 2**.

**Table 2:** Phylogenetic group name (from **Figure 1**), number of individuals within each group (*N*) and the number of diagnostic SNPs (*D_snps_*).

Group	*N*	*D_snps_*
EIEC/EHEC/EAEC	12	6
EIEC large	16	0
EIEC small	3	31
ExPEC	6	71
SB/SD large	38	7
SB/SD small	15	21
SD serotype 1	3	1
SD serotype 10	3	37
SD serotype 8/EHEC/EPEC	10	1
SF	33	34
SS	26	45

Total	165	254

To illustrate the importance of using a genetically diverse set of genomes for the development of molecular epidemiological markers, we performed *in silico* analyses of primer sequence identities using BLAST searches for each primer against the full set (169) of genomes with a sequence identity of 95% (one base pair difference per primer) or higher for primers from (Sahl et al., 2015) or 92% and higher for primers from (Pavlovic et al., 2011; **Supplementary Figures S5** and **S6**). We predict that these primers would not accurately distinguish between the phylogenetic groups determined by Sahl et al. (2015) or between *Shigella* and EIEC genomes, as suggested by Pavlovic et al. (2011; **Supplementary Figures S5** and **S6**).

## Discussion

To the best of our knowledge, this study represents the most comprehensive phylogeny of *Shigella* and EIEC to date. Unlike previous studies exploring the molecular relationships between *E. coli* and *Shigella* (Pupo et al., 2000; Lan and Reeves, 2002; Escobar-Páramo et al., 2003, 2004; Lan et al., 2004; Touchon et al., 2009; Sims and Kim, 2011; Zhang and Lin, 2012; Gardner and Hall, 2013; Zuo et al., 2013; Sahl et al., 2014, 2015), we used a large number and diversity of *Shigella* and EIEC genomes, including the recently discovered SB serotypes 19 and 20 and SD serotype 15, and performed genomic-scale phylogenetic analyses. The phylogeny together with the population structure analyses and the clustering of translated gene abundance profiles suggest that *Shigella* and EIEC evolved independently (**Figures 1–3**). Due to the polyphyly observed for EIEC, EIEC as a group cannot be considered as the ancestor to *Shigella* although some EIEC lineages may be the ancestor to *Shigella* (**Figure 1**). Interestingly, the phylogeny obtained is similar to the ones constructed using multi locus genotype data and other inference methods (i.e., neighbor-joining; Pupo et al., 2000; Lan and Reeves, 2002; Escobar-Páramo et al., 2003; Lan et al., 2004).

### Incongruence between Phylogeny and Taxonomy

A few studies have concluded that *Shigella* arose from a single common ancestor (or monophyletically; Escobar-Páramo et al., 2003; Zuo et al., 2013). This conclusion likely comes from phylogenetic analyses conducted with a limited diversity of *Shigella* strains and serotypes and EIEC isolates. Analyses that include a broader diversity of strains support a hypothesis of multiple origins (Pupo et al., 2000; Lan and Reeves, 2002; Lan et al., 2004; Sahl et al., 2015). Although many topological characteristics of our SNP-based phylogeny, such as the polyphyly of SB/SD, have been identified previously (Pupo et al., 2000; Lan and Reeves, 2002; Escobar-Páramo et al., 2003, 2004; Lan et al., 2004; Sahl et al., 2015), we clearly show that *Shigella* and EIEC genomes originated from multiple independent events. Similarly, the grouping of SF serotype 6 near SB serotypes 2, 4, and 14 indicates that, despite being called SF, they are part of the SB/SD large lineage (Pupo et al., 2000; Lan et al., 2004). As expected from previous studies that link SB serotype 13 to *E. albertii* (Pupo et al., 2000; Lan and Reeves, 2002; Hyma et al., 2005), our SB serotype 13 representative genome clusters outside of *E. coli*, EIEC and *Shigella* groups where it appears as the base of the phylogeny on an exceptionally long branch.

When considering EIEC specifically, our results are in agreement with those of Lan et al. (2004) where O124, O152, and O135 serotypes cluster together and O136, O28ac, O164, and O29 cluster together. Similarly, we observed, that EIEC serotype O112ac clustered near SB serotype 12 and SD serotype 2, and identified only five core SNP differences between EIEC serotype O112ac and SD serotype 2(2) **Figures 1–3**).

One topological difference between our phylogeny and previous phylogenies is the clustering of SB serotype 12. In our phylogenetic analyses (**Figure 1**), SB serotype 12 clusters in the SB/SD small cluster as opposed to clustering with SF strains in trees constructed by Pupo et al. (2000) and Lan et al. (2004). Our kSNP analyses reveal that there are only eight core SNP differences between SB serotype 12 and SD serotype 2(1). However, clustering of the translated gene abundance matrix shows that SB serotype 12 clusters by itself, away from any isolates it clusters near in the phylogeny (**Figure 3**). This suggests that SB serotype 12 may have a unique genetic history requiring additional analyses.

Given that we did not remove homoplastic SNPs based on the phylogenetic results, we can infer the degree of admixture (perhaps due to recombination) among the samples based on the STRUCTURE results. In general, both the clustering at *k* = 6 and 11 show only a few samples to have SNP profiles that suggest admixture with other distinct groups. Also from the STRUCTURE analyses, we see that hybrid strains within SB, SD, and SS lineages may be rare. An exception is SB serotype 9 (**Figure 3**) and, similar to SB serotype 12 discussed above, the hierarchical clustering of the translated gene abundance matrix shows it clustering distantly from strains it clusters near in the phylogeny. It would be interesting to further investigate a range of SB serotype 9 isolates to determine if this pattern is common and represents a transitional strain.

While we did not specifically investigate the evolutionary history of the invasion plasmid, our data do not support the hypothesis proposed by Escobar-Páramo et al. (2003) that the invasion plasmid was transferred before the evolution of *Shigella* and EIEC lineages. Our phylogeny and the DISTRUCT diagram (**Figures 1** and **2**) suggest that EIEC cluster with non-invasive *E. coli* genomes that do not possess the invasion plasmid implying that the transfer of the invasion plasmid did not precede a monophyletic evolution of *Shigella* and EIEC.

### Importance of Sampling Diverse Genetic Lineages

Our study underscores the importance of including a diverse collection of *Shigella* and EIEC genomes into phylogenetic studies that examine *Shigella*, as we were able to make a number of novel findings with high confidence. For example, EIEC strains appear to have a greater genetic diversity than previously believed, with EIEC strains clustering near non-invasive *E. coli* strains (**Figure 1**). For this reason, the inclusion of a range of EIEC strains for developing diagnostic tools is essential for accurate and clinically relevant identification, as well as for outbreak detection. When genetic diversity is not a component of investigations for diagnostic purposes, markers may not be useful. One example is a recent study that presents diagnostic markers for PCR detection of *Shigella* (Sahl et al., 2015), yet the primers for these markers do not discriminate between *Shigella* and EIEC when a larger genetic diversity is considered (**Supplementary Figure S5**). While another study included 11 EIEC strains (Pavlovic et al., 2011), their primers and probes cannot accurately distinguish between *Shigella* and EIEC (**Supplementary Figure S6**).

Single nucleotide polymorphisms markers are a useful genotyping/molecular epidemiological typing method because they are considered relatively genetically stable and not likely to change, to such a degree that classification tools are built based on SNPs (Larkeryd et al., 2014). Another asset is that a nucleotide should always be present at the SNP position, reducing the number of false negatives from presence/absence-type gene markers. SNP detection methods are also considered excellent for their discriminatory power, reproducibility and ability to be used in a high-throughput capacity (Hallin et al., 2012). With these advantages in mind, we identified multiple SNPs for the phylogenetic groups (except EIEC large), which offer researchers multiple opportunities for optimizing primer design and confirming positive results. Our inability to identify diagnostic SNP markers for the EIEC large cluster suggests that a greater diversity of EIEC isolates would be needed for markers (**Supplementary Table S3**). The lower bootstrap support (0.61) for the EIEC large cluster (**Figure 1**) is consistent with a need for additional genomes with greater genetic diversity.

Analyses looking for the presence/absence of core genes that were specific to each cluster yielded no such genes. This is in agreement with another study that did not identify *Shigella*-specific genes that were distinct from *E. coli* using orthologous genes from pan-genomes (Gordienko et al., 2013). As the authors and our data suggest, phylogenetic evidence points toward *Shigella* belonging to the *E. coli* genus and thus these groups are likely sharing the same pool of genes. In summary, the polyphyletic nature of the *Shigella* and *E. coli* groups and putative taxonomy makes the strategy of identifying specific genes to these groups difficult.

The clustering based on the abundance matrix of translated genes is not strictly congruent with the topology inferred from the phylogenetic analyses using the SNP data (**Figures 1** and **3**). However, most of the incongruence is among clades rather than the membership of individuals to specific clades. For example, all but one of the individuals belonging to the SF, SB/SD large, and SS clusters are not found grouped together in the trees based on the SNP and gene abundance data but the relationships among those clades does differs (**Figures 1** and **3**). Overall, we find support that gene content/abundance carries a similar evolutionary signal as that contained in SNPs. For example, there is an appreciable amount of resolution and fidelity to the relationships depicted in the phylogeny using the hierarchically clustered distance matrix of predicted protein homologs for clusters of genomes from SF, SS, SB/SD large clusters (**Figure 3**). These clusters have significant AU values and strong bootstrap support. However, differences do exist between the methods, which may be the result of unresolved basal relationships and/or unique isolate outliers (such as EIEC O96:H16, SB serotypes 9 and 12). It is also possible that the gene abundance analyses are capturing a stronger signal from recombination and mobile elements than would be present in the core SNP matrix. A similar incongruence was observed in a very limited number of *Shigella* and *E. coli* genomes between phylogenies based on core SNPs and using BLAST derived coding sequences (CDSs; Sahl et al., 2014). Some degree of incongruence is to be expected due to gene histories being linked but different from species histories (Szöllosi et al., 2015). For example, studies of SS and SD provide evidence that these lineages are undergoing selection for drug and multidrug resistance and we also observed a pattern of clustering of antibiotic resistance-related genes that are linked to phylogeny but also may have individual gene histories (**Supplementary Figure S4**; Holt et al., 2012; Rohmer et al., 2014).

## Conclusion

There is a growing acknowledgment that microbial taxonomy should be based on a more comprehensive and exhaustive survey of genomes (Rosselló-Móra and Amann, 2015; Thompson et al., 2015). Current problematic taxonomic designations are common throughout microbial taxonomy (Rosselló-Móra and Amann, 2015; Thompson et al., 2015 and references within). In the case of *Shigella*, genomic evidence supporting the change of taxonomic designations is well-established (Pupo et al., 2000; Lan and Reeves, 2002; Escobar-Páramo et al., 2003, 2004; Lan et al., 2004; Gardner and Hall, 2013; Sahl et al., 2014, 2015). Based on these studies and the analyses conducted herein, there is a large body of evidence that the *Shigella* genus should be moved back within the species *E. coli*. Furthermore, we suggest that *Shigella* should be classified as EIEC and the serotypes renamed using the common O antigen naming. *Shigella* serotypes are based upon O antigens many of which are identical or nearly identical to existing *E. coli* O antigens (with the exception of *S. sonnei*; Liu et al., 2008). The existence of two separate nomenclatures is redundant and confusing. We are repeating a long established call to reduce confusion and promote the understanding of accurate evolutionary relationships of *Shigella* and *E. coli* (Lan and Reeves, 2002; Chaudhuri and Henderson, 2012). While we believe that taxonomic designations that more accurately reflect genetic relationships can improve outbreak characterization and communication in the long-term, taxonomic revisions are difficult and some may consider that revisions pose risks for public health in the more immediate time frame. We support the growing recognition of the value behind systematic species or genome similarity assignments for all players involved in real-time epidemics (Marakeby et al., 2014; Varghese et al., 2015; Weisberg et al., 2015). In the absence of universal genome-based classification and naming systems, our results provide support for reconsidering the current taxonomic placement and naming of *Shigella* species.

## Author Contributions

EP generated sequence data. EP and JP performed analyses. EP, JP, and RB interpreted results and wrote the manuscript. RB conceived the project. All authors read and approved the final manuscript.

## Conflict of Interest Statement

The authors declare that the research was conducted in the absence of any commercial or financial relationships that could be construed as a potential conflict of interest.

## Footnotes

1 http://www.ncbi.nlm.nih.gov/Traces/sra/sra.cgi?cmd=show&f=software&m=software&s=software

2 https://cge.cbs.dtu.dk/services/

## Supplementary Material

FIGURE S1 A maximum-likelihood (ML) phylogeny of *Shigella*, enteroinvasive *E. coli* (EIEC), non-invasive *E. coli* strains and *Salmonella* outgroup based on 2,348 SNPs present in all genomes using the kSNP program (Gardner and Hall, 2013). The ML tree was generated using GARLI v. 2.0.1019 (Zwickl, 2006) under the GTR + I + Γ model and other default settings. Trees were visualized with Figtree v. 1.3 (Rambaut and Drummond, 2009). The best tree was chosen from 100 runs of the data set and bootstrap values (1,000 iterations) are reported above each node. Bootstrap values <80% were not shown.

FIGURE S2 Histograms of the pairwise distances of core SNP differences between genome pairs for the SNP-based phylogenies **(A)** without the Salmonella outgroup and **(B)** with the *Salmonella* outgroup.

FIGURE S3 Reordered STRUCTURE results for *S. flexneri* genomes from analyses performed with 11 SNP groups (right) corresponding to the phylogenetic cluster in **Figure 1** (left).

FIGURE S4 Hierarchical clustering of antibiotic resistance related genes. Red values on dendrogram represent unbiased *p*-values determined by Pvclust package in R. The dendrogram was generated using the correlation distance method and the average linkage method.

FIGURE S5 BLAST alignment of primers, described by Sahl et al. as specific for Shigella phylogenetic groups (Sahl et al., 2015), with genomes used in this study. A blue cell for a particular genome indicates that both primers of the pair aligned to 95% or greater sequence identity and should therefore hybridize to yield a PCR product. The phylogenetic group designation assigned by Sahl et al. is noted next to the cluster designations we observed with these genomes.

FIGURE S6 *In silico* alignment of primer-probe sets described by Pavlovic et al. (2011) with genomes used in this study using BLAST. The *lacY* set was supposed to differentiate between *Shigella* (absent) and EIEC (present), while the *uidA* set was intended to be a positive control (present in both). BLAST identities of 92% or higher are shown with blue cells. Although PCR products are expected from a particular genome if both cells corresponding to the forward and reverse primers are highlighted in blue, the real-time PCR assay (Pavlovic et al., 2011) also require the respective probe to hybridize efficiently and therefore the respective cell to be highlighted in blue in the figure.

TABLE S1 Strain information includes NCBI identifier (SRA#), Tree label/Strain designation, genus and species with serotype, O or H antigens, additional strain identifiers and reference for source of genomes.

TABLE S2 Full list of diagnostic SNPs for *Shigella* and EIEC phylogenetic clusters. Includes phylogenetic cluster name, 21 bp sequence of region containing diagnostic SNP with ambiguous SNP state represented by “.”, diagnostic SNP state of cluster, position in the NCBI annotated reference genome (SD serotype 1, CP000034), gene name (“NA” if intergenic), functional gene product (“NA” if intergenic), COG identifier and reference genome (CP000034) locus tag.

TABLE S3 Assembly statistics and genome metrics calculated by the Quast program. Includes Tree label/Strain designation, NCBI SRA accession number, number of contigs greater or equal to 1,000 bp (# contigs (≥1,000 bp)), number of contigs greater or equal to 0 bp ((# contigs (≥0 bp), total length of contigs greater or equal to 1,000 bp (Total length (≥1,000 bp)), total length of contigs greater or equal to 0 bp (Total length (≥0 bp)), number of contigs, largest contig (bp), total length of all contigs, percent GC content and number of N’s per 100 kbp.
