# Corrigendum: Phylogenetic Analyses of *Shigella* and Enteroinvasive *Escherichia coli* for the Identification of Molecular Epidemiological Markers: Whole-Genome Comparative Analysis Does Not Support Distinct Genera Designation

**DOI:** 10.3389/fmicb.2017.02598

**Published:** 2018-01-09

**Authors:** Emily A. Pettengill, James B. Pettengill, Rachel Binet

**Affiliations:** ^1^Division of Microbiology, Office of Regulatory Science, U.S. Food and Drug Administration, Center for Food Safety and Applied Nutrition, College Park, MD, United States; ^2^Division of Public Health Informatics and Analytics, Office of Analytics and Outreach, U.S. Food and Drug Administration, Center for Food Safety and Applied Nutrition, College Park, MD, United States

**Keywords:** *Shigella*, enteroinvasive *E. coli* (EIEC), phylogeny, whole genome sequencing, classification, epidemiological markers

In the original article, there was a mistake in Table 1 as published. Our collection stock of EIEC-O152 (1) contained low level of ExPEC-O25:H16 which was sequenced in the study instead of EIEC. The corrected Table 1 appears below.

**Table 1:** Number of bacterial isolates and serotypes.

**Tree label**	**Description**	**Isolates**	**Serotypes**
EIEC	Enteroinvasive *E. coli*	32	15
EAEC	Enteroaggregative *E. coli*	3	3
STEC	Shiga-toxin producing *E. coli*	1	1
ExPEC	Extraintestinal *E. coli*	6	3
EPEC	Enteropathogenic *E. coli*	3	2
EHEC	Enterohemorrhagic *E. coli*	5	5
*E. fergusonii*	*E. fergusonii*	2	1
SD	*Shigella dysenteriae*	23	14
SF	*Shigella flexneri*	36	6
SB	*Shigella boydii*	32	20
SS	*Shigella sonnei*	26	1
*S. enterica*	*Salmonella enterica*	2	1
	Total	171	72

The ExPEC cluster contains the ExPEC-O25:H16 instead of the one EIEC isolate but this cluster was not discussed in the original manuscript. The NCBI accession has been updated as well.

Labels for EIEC-O152 (1) were modified to ExPEC-O25:H16 on the phylogenetic trees displayed in Figures 1, 3, and in Supplementary Figures 1, 4–6, and in Supplementary Tables 1, 3.

**Figure 1:**
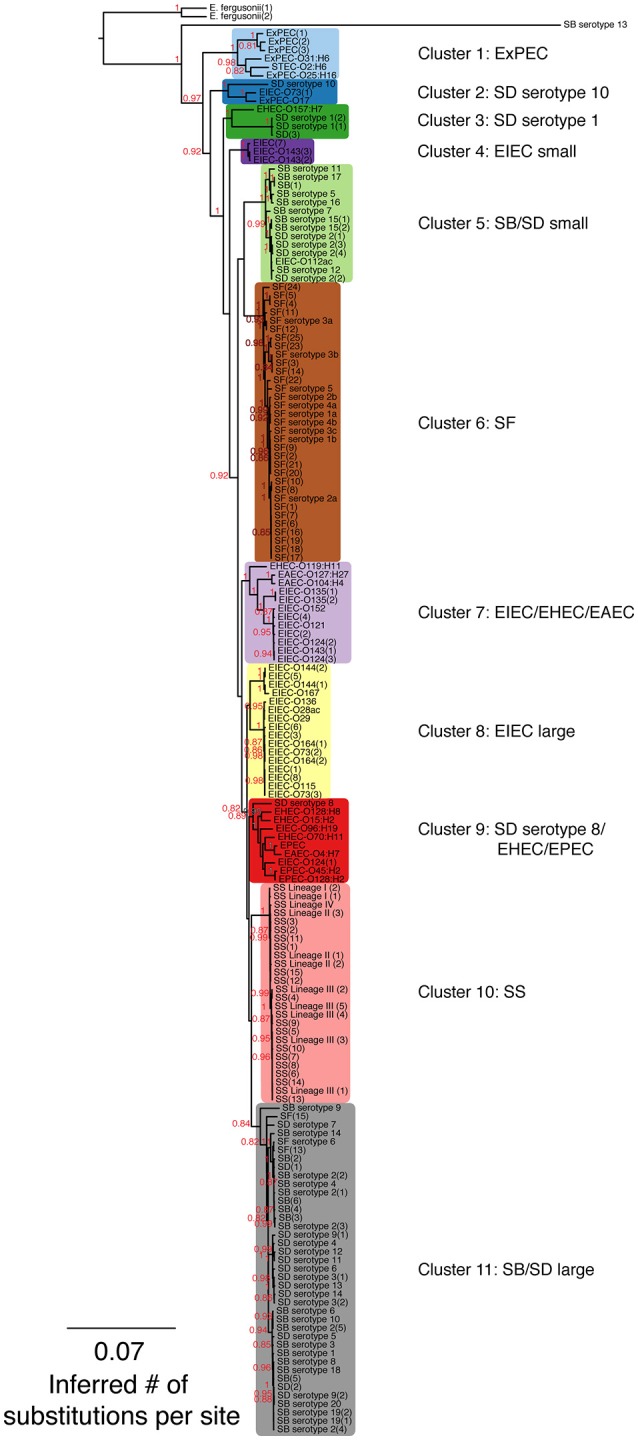
A maximum-likelihood (ML) phylogeny of *Shigella*, enteroinvasive *E. coli* (EIEC) and non-invasive *E. coli* strains based on 7,062 core SNPs using kSNP (Gardner and Hall, 2013). The ML tree was generated using GARLI v. 2.0.1019 under the GTR + I + Γ model and other default settings (Zwickl, 2006). Trees were visualized with Figtree v. 1.3.1 (Rambaut and Drummond, 2009). The best tree was chosen from 1,000 runs of the data set and bootstrap values (1,000 iterations) are reported above each node. Bootstrap values <80% are not shown. A tree that includes the Salmonella outgroup can be found in Supplementary Figure 1.

**Figure 3:**
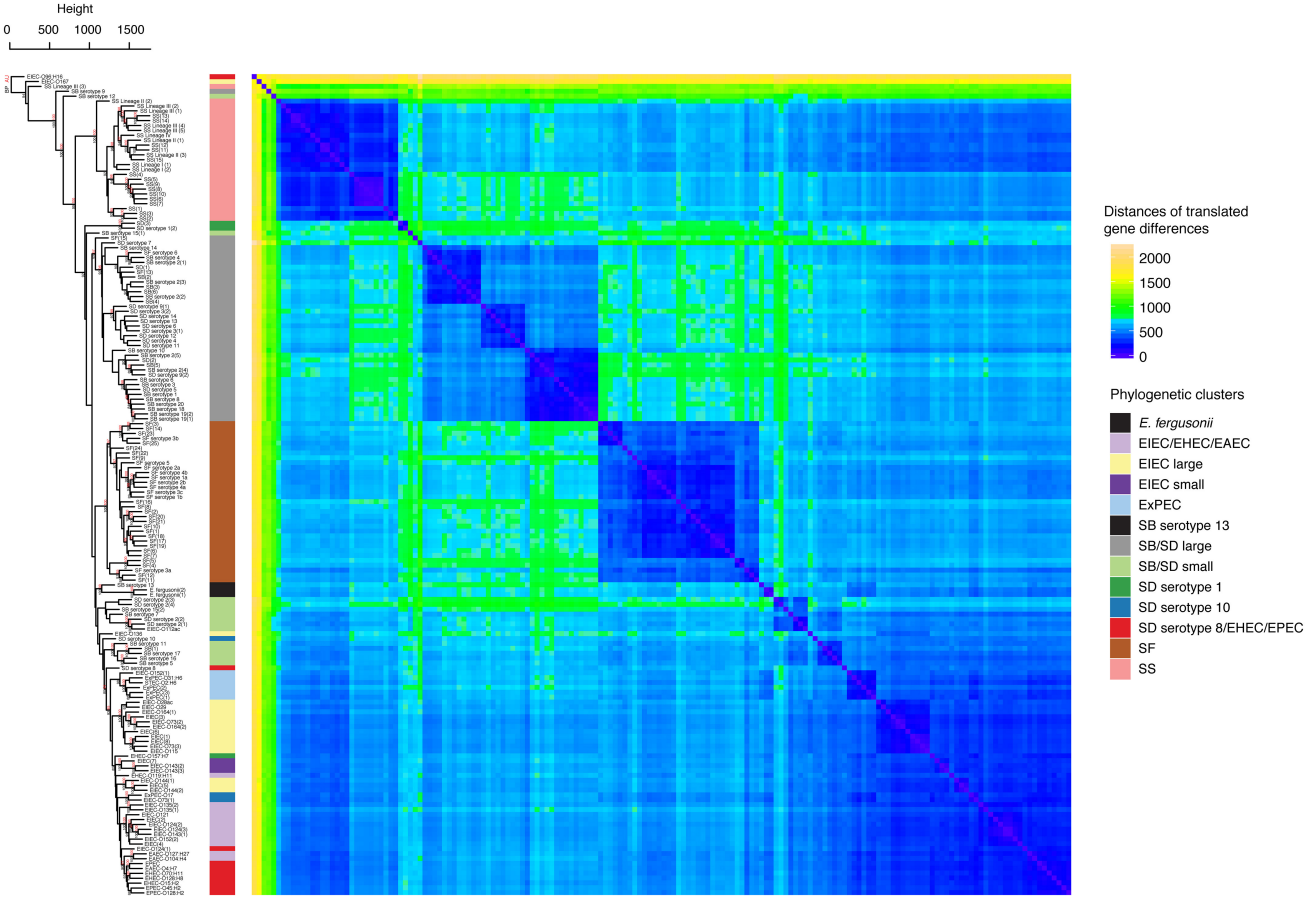
Hierarchical clustering and heat map illustrating the differences in predicted protein homologs between genomes. Manhattan distances were calculated from a pairwise abundance matrix of 3,777 predicted protein homologs that were identified using the default BLASTP bidirectional best hit approach (75% amino acid sequence coverage, 1e-05 E-value and 60% sequence identity) within the program GET_HOMOLOGUES (Contreras-Moreira and Vinuesa, 2013). Only genes shared by at least two samples were included. Blue cells on the heat map indicate that genomes share more similar genes. The dendrogram on y-axis indicates hierarchical clustering of the abundance matrix using the average linkage method and Manhattan distances with bootstrap probabilities (BP, only values of ≥80 shown in black) and approximately unbiased *p*-values (AU, only values of ≥95 shown in red) from 10,000 replicates. The phylogenetic group of each genome from Figure 1 is represented as a colored bar in between the dendrogram and the heat map.

Additionally, the text in the Phylogeny subsection in Results, first paragraph, should be written as:

One hundred and seventy-one genomes were selected to encompass a large selection of EIEC strains and represent the diversity of the *Shigella* genus. Genomes from 35 isolates were in-house sequenced draft genomes while 136 were available in public databases (Supplementary Table 1). We used 23 isolates of SD, including a minimum of 14 serotypes, 36 SF isolates, including at least six serotypes, 32 SB isolates, covering all 20 serotypes, 26 SS isolates, **32** EIEC isolates with 15 different serotypes, **18** isolates of non-invasive *E. coli* composed of 14 different serotypes, two isolates of *E. fergusonii*. The genomes of two *Salmonella* isolates were used for an outgroup (Table 1).

In the original article, there was a mistake in Table 2 as published. A coding mistake led to incorrect identification of lineage-specific SNPs. We reported 404 diagnostic SNPs, but the correct count is 254. The corrected Table 2 appears below and Supplementary Table 2 with the sequences of the regions containing the diagnostic SNPs has been modified.

**Table 2:** Phylogenetic group name (from Figure 1), number of individuals within each group (*N*) and the number of diagnostic SNPs (*D*_*snps*_).

**Group**	***N***	***D_*snps*_***
EIEC/EHEC/EAEC	12	6
EIEC large	16	0
EIEC small	3	31
ExPEC	6	71
SB/SD large	38	7
SB/SD small	15	21
SD serotype 1	3	1
SD serotype 10	3	37
SD serotype 8/EHEC/EPEC	10	1
SF	33	34
SS	26	45
Total	165	254

The abstract should read “Lastly, we identified a panel of **254** single nucleotide polymorphism (SNP) markers specific to each phylogenetic cluster for more accurate identification of *Shigella* and EIEC.” Similarly, the second line in the Lineage-Specific SNP Identification and Evaluation of Previously Described Molecular Assays for the Differentiation of *Shigella* and EIEC subsection in Results should read: “From 7,062 core SNPs, we found **254** SNP positions that were diagnostic for each of the clusters (Supplementary Table S2).”

The authors apologize for these errors and state that this does not change the scientific conclusions of the article in any way.

The original article has been updated.

## Conflict of interest statement

The authors declare that the research was conducted in the absence of any commercial or financial relationships that could be construed as a potential conflict of interest.

## Supplementary material

Supplementary Figure 1 A maximum-likelihood (ML) phylogeny of *Shigella*, enteroinvasive *E. coli* (EIEC), non-invasive *E. coli* strains and *Salmonella* outgroup based on 2,348 SNPs present in all genomes using the kSNP program (Gardner and Hall, 2013). The ML tree was generated using GARLI v. 2.0.1019 (Zwickl, 2006) under the GTR + I + Γ model and other default settings. Trees were visualized with Figtree v. 1.3 (Rambaut and Drummond, 2009). The best tree was chosen from 100 runs of the data set and bootstrap values (1,000 iterations) are reported above each node. Bootstrap values <80% were not shown.

Supplementary Figure 4 Hierarchical clustering of antibiotic resistance related genes. Red values on dendrogram represent unbiased *p*-values determined by Pvclust package in R. The dendrogram was generated using the correlation distance method and the average linkage method.

Supplementary Figure 5 BLAST alignment of primers, described by Sahl et al. as specific for Shigella phylogenetic groups (Sahl et al., 2015), with genomes used in this study. A blue cell for a particular genome indicates that both primers of the pair aligned to 95% or greater sequence identity and should therefore hybridize to yield a PCR product. The phylogenetic group designation assigned by Sahl et al. is noted next to the cluster designations we observed with these genomes.

Supplementary Figure 6 *In silico* alignment of primer-probe sets described by Pavlovic et al. (2011) with genomes used in this study using BLAST. The *lacY* set was supposed to differentiate between *Shigella* (absent) and EIEC (present), while the *uidA* set was intended to be a positive control (present in both). BLAST identities of 92% or higher are shown with blue cells. Although PCR products are expected from a particular genome if both cells corresponding to the forward and reverse primers are highlighted in blue, the real-time PCR assay (Pavlovic et al., 2011) also require the respective probe to hybridize efficiently and therefore the respective cell to be highlighted in blue in the figure.

Supplementary Table 1 Strain information includes NCBI identifier (SRA#), Tree label/Strain designation, genus and species with serotype, O or H antigens, additional strain identifiers and reference for source of genomes.

Supplementary Table 2 Full list of diagnostic SNPs for *Shigella* and EIEC phylogenetic clusters. Includes phylogenetic cluster name, 21 bp sequence of region containing diagnostic SNP with ambiguous SNP state represented by “.”, diagnostic SNP state of cluster, position in the NCBI annotated reference genome (SD serotype 1, CP000034), gene name (“NA” if intergenic), functional gene product (“NA” if intergenic), COG identifier and reference genome (CP000034) locus tag.

Supplementary Table 3 Assembly statistics and genome metrics calculated by the Quast program. Includes Tree label/Strain designation, NCBI SRA accession number, number of contigs greater or equal to 1,000 bp (# contigs (≥1,000 bp)), number of contigs greater or equal to 0 bp ((# contigs (≥0 bp), total length of contigs greater or equal to 1,000 bp (Total length (≥1,000 bp)), total length of contigs greater or equal to 0 bp (Total length (≥0 bp)), number of contigs, largest contig (bp), total length of all contigs, percent GC content and number of N's per 100 kbp.

