# Supplementary figures and images for: Corrigendum: Phylogenetic Analyses of Shigella and Enteroinvasive Escherichia coli for the Identification of Molecular Epidemiological Markers: Whole-Genome Comparative Analysis Does Not Support Distinct Genera Designation
Source: Front Microbiol. 2018 Jan 9;8:2598. doi: 10.3389/fmicb.2017.02598 (PMC5767330; doi:10.3389/fmicb.2017.02598)

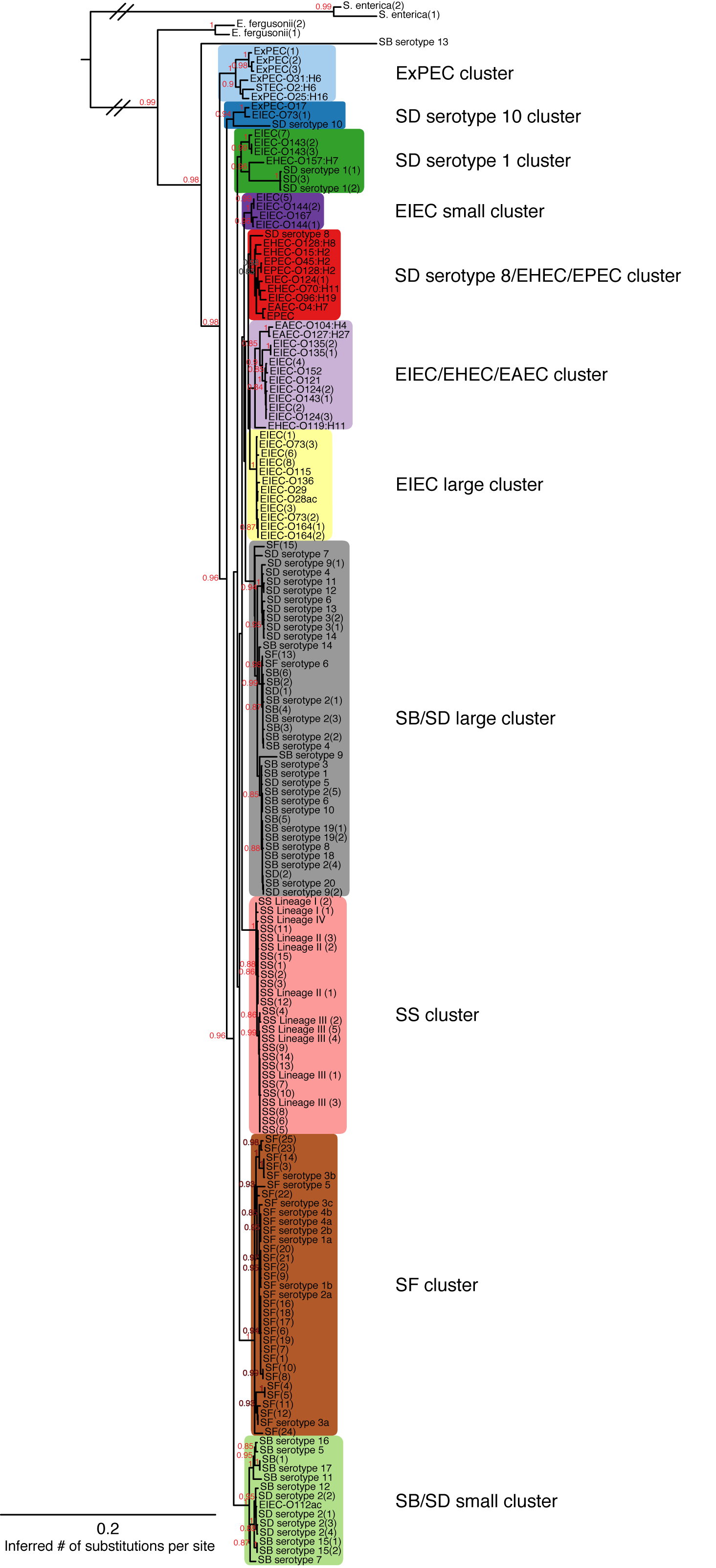

Supplement: Supplementary Figure 1 — A maximum-likelihood (ML) phylogeny of Shigella, enteroinvasive E. coli (EIEC), non-invasive E. coli strains and Salmonella outgroup based on 2,348 SNPs present in all genomes using the kSNP program (Gardner and Hall, 2013). The ML tree was generated using GARLI v. 2.0.1019 (Zwickl, 2006) under the GTR + I + Γ model and other default settings. Trees were visualized with Figtree v. 1.3 (Rambaut and Drummond, 2009). The best tree was chosen from 100 runs of the data set and bootstrap values (1,000 iterations) are reported above each node. Bootstrap values <80% were not shown. [file Image1.TIF]

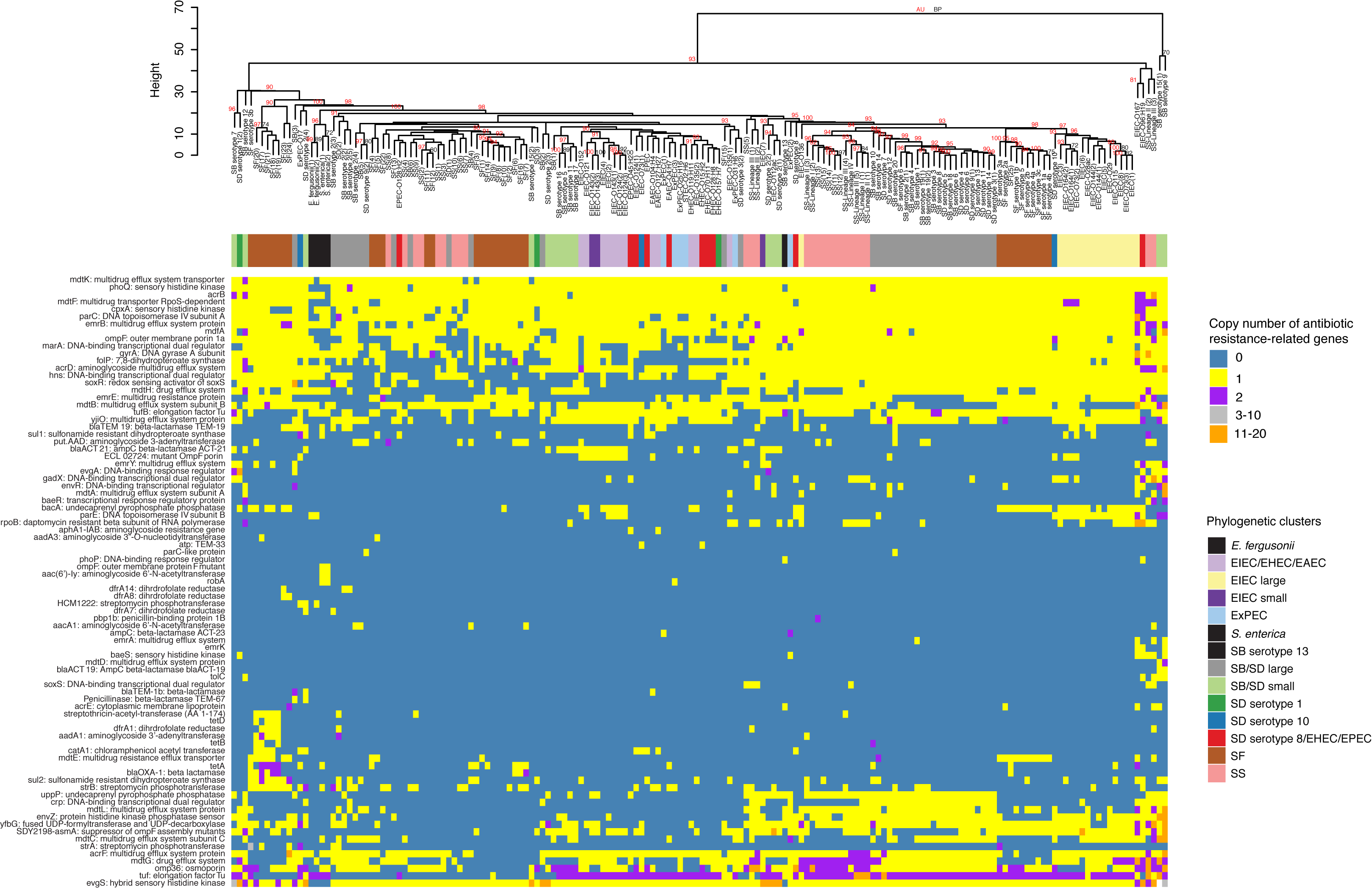

Supplement: Supplementary Figure 4 — Hierarchical clustering of antibiotic resistance related genes. Red values on dendrogram represent unbiased p-values determined by Pvclust package in R. The dendrogram was generated using the correlation distance method and the average linkage method. [file Image4.TIF]

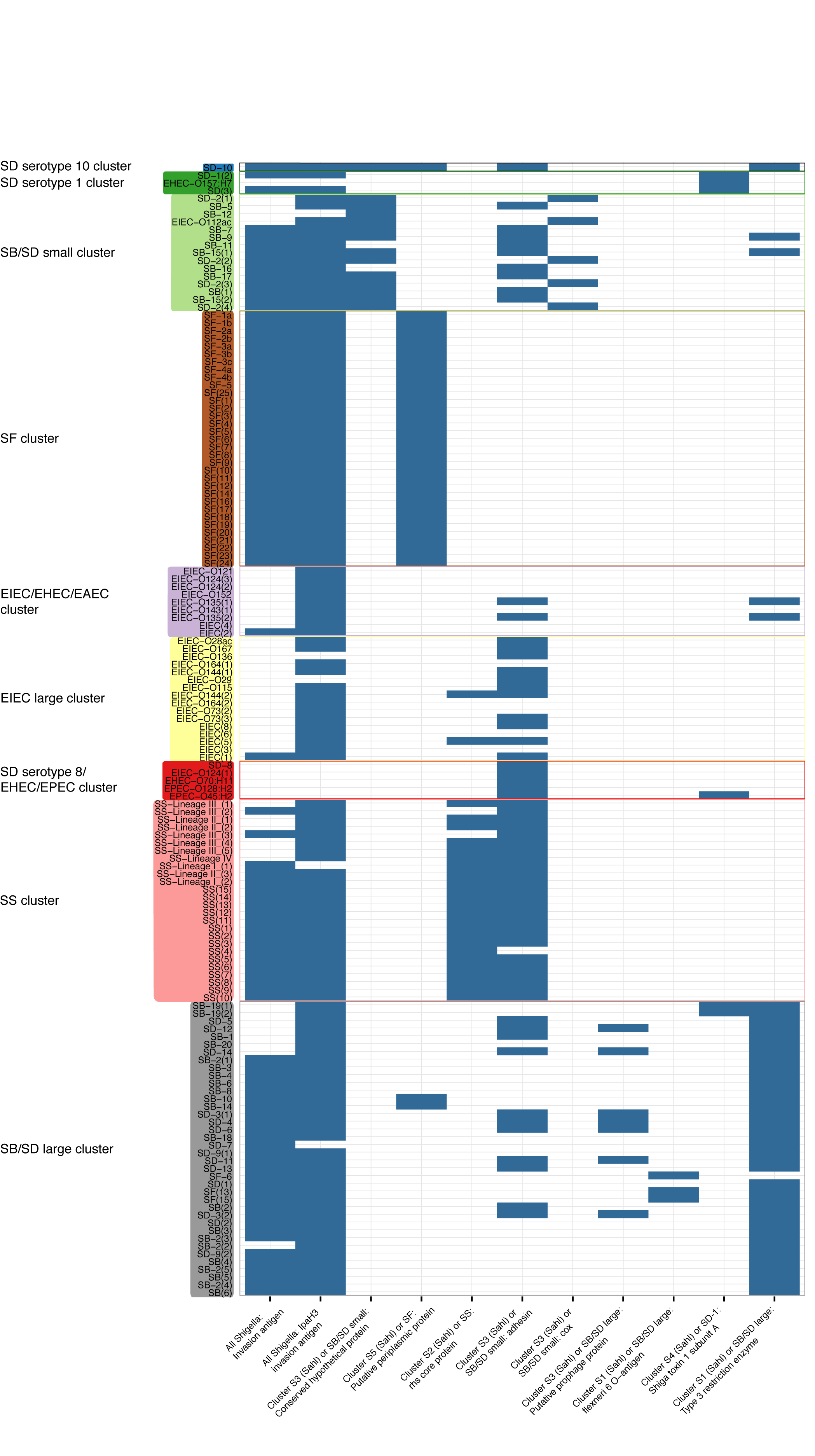

Supplement: Supplementary Figure 5 — BLAST alignment of primers, described by Sahl et al. as specific for Shigella phylogenetic groups (Sahl et al., 2015), with genomes used in this study. A blue cell for a particular genome indicates that both primers of the pair aligned to 95% or greater sequence identity and should therefore hybridize to yield a PCR product. The phylogenetic group designation assigned by Sahl et al. is noted next to the cluster designations we observed with these genomes. [file Image5.TIF]

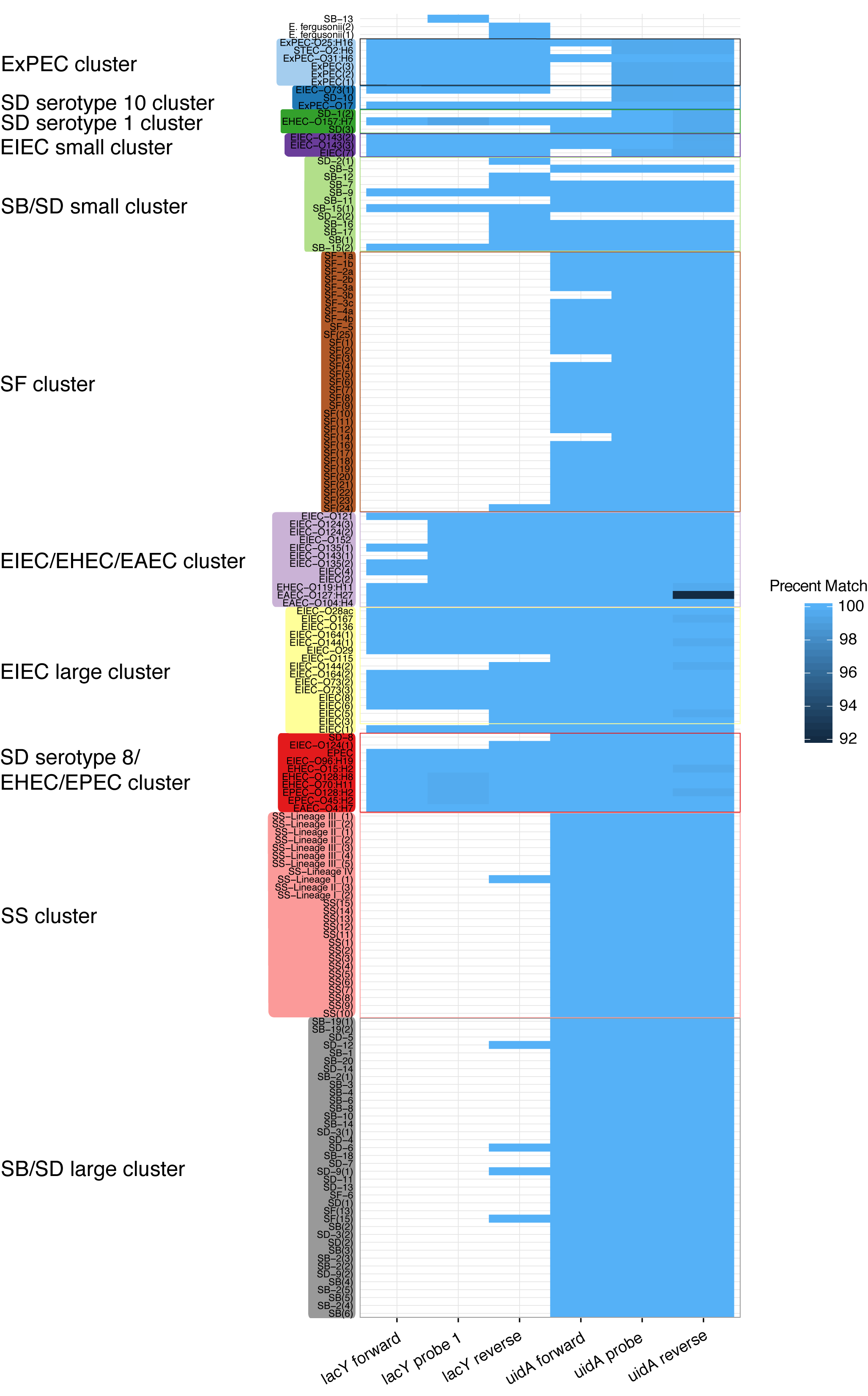

Supplement: Supplementary Figure 6 — In silico alignment of primer-probe sets described by Pavlovic et al. (2011) with genomes used in this study using BLAST. The lacY set was supposed to differentiate between Shigella (absent) and EIEC (present), while the uidA set was intended to be a positive control (present in both). BLAST identities of 92% or higher are shown with blue cells. Although PCR products are expected from a particular genome if both cells corresponding to the forward and reverse primers are highlighted in blue, the real-time PCR assay (Pavlovic et al., 2011) also require the respective probe to hybridize efficiently and therefore the respective cell to be highlighted in blue in the figure. [file Image6.TIF]
